# Disease Profile and Achievement of Therapeutic Goals in a Modern, Nationwide Cohort of 923 Patients with Psoriatic Arthritis

**DOI:** 10.31138/mjr.301223.dpa

**Published:** 2023-12-30

**Authors:** George E. Fragoulis, Charalampos Papagoras, Sousana Gazi, Evangelia Mole, Michael Krikelis, Paraskevi V. Voulgari, Evripidis Kaltsonoudis, Nikolaos Koletsos, Pelagia Katsimpri, Dimitrios Boumpas, Dimitrios Katsifis, Nikolaos Kougkas, Theodoros Dimitroulas, Petros P. Sfikakis, Maria G. Tektonidou, Chrysoula Gialouri, Dimitrios P. Bogdanos, Theodora Simopoulou, Christos Koutsianas, Eugenia Mavrea, Gkikas Katsifis, Konstantinos Kottas, Maria Konsta, Matthoula Tziafalia, Evangelia Kataxaki, Eleni Kalavri, Kalliopi Klavdianou, Eleftheria P. Grika, Charalampos Sfontouris, Dimitrios Daoussis, George Iliopoulos, Ilias Bournazos, Dimitrios Karokis, Konstantinos Georganas, Dimos Patrikos, Dimitrios Vassilopoulos

**Affiliations:** 1 Joint Academic Rheumatology Program, First Department of Propaedeutic and Internal Medicine, National and Kapodistrian University of Athens, Athens, Greece,; 2 First Department of Internal Medicine, University Hospital of Alexandroupolis, Democritus University of Thrace, Alexandroupolis, Greece,; 3 Department of Rheumatology, KAT Hospital, Athens, Greece,; 4 Department of Rheumatology, School of Health Sciences, Faculty of Medicine, University of Ioannina, Ioannina, Greece,; 5 Joint Academic Rheumatology Program, 4^th^ Department of Internal Medicine, Attikon University Hospital, National and Kapodistrian University of Athens Medical School, Athens, Greece,; 6 4^th^ Department of Medicine, Aristotle University, Thessaloniki, Greece,; 7 Department of Rheumatology and Clinical Immunology, University of Thessaly, Larissa, Greece,; 8 Joint Academic Rheumatology Program, Clinical Immunology, Rheumatology Unit, 2^nd^ Department of Medicine and Laboratory, National and Kapodistrian University of Athens Medical School, General Hospital of Athens “Hippokration”, Athens, Greece,; 9 Rheumatology Clinic, Naval Hospital of Athens, Athens, Greece,; 10 Rheumatology Unit, Sismanoglio Hospital, Athens, Greece,; 11 Rheumatology Department, General Hospital Elefsinas Thriaseio, Athens, Greece,; 12 Department of Rheumatology, “Asklepieion” General Hospital, Athens, Greece,; 13 Department of Rheumatology, Evaggelismos Athens General Hospital, Athens, Greece,; 14 Department of Rheumatology, Patras University Hospital, University of Patras Medical School, Patras, Greece,; 15 Private practice, Athens, Greece

**Keywords:** psoriatic arthritis, comorbidities, bDMARDs, minimal disease activity, registry

## Abstract

**Background:**

Psoriatic arthritis (PsA) is a heterogenous chronic inflammatory disease affecting skin, joints, entheses, and spine with various extra-musculoskeletal manifestations and comorbidities. The reported patient, disease and treatment characteristics in the modern therapeutic era are limited.

**Methods:**

In this cross-sectional, multi-centre, nationwide study, we recorded the demographic, clinical, and therapeutic characteristics as well as the comorbidities of patients with PsA seen for 1 year (1/1/2022-31/12/2022).

**Results:**

923 patients (55% females) with a median (IQR) age of 57 (48-65) years and a mean disease duration of 9.5 years were enrolled. Family history of psoriasis and PsA was noted in 28.3% and 6.3%, respectively. Most patients had limited psoriasis (BSA<3: 83%) while enthesitis, dactylitis, nail and axial involvement reported in 48.3%, 33.2%, 43% and 25.9% of patients, respectively. Regarding comorbidities, approximately half of patients had dyslipidaemia (42%) or hypertension (45.4%), 36.8% were obese and 17% had diabetes while 22.7% had a depressive disorder. Overall, 60.1% received biologics and among them more patients treated with anti-IL-17 or -12/23 agents were on monotherapy (64.2%) compared to those on TNFi monotherapy (49.4%, p=0.0001). The median PsA activity as assessed by the DAPSA score was 6 (IQR: 2.3 – 13.1) with 46% of patients reaching minimal disease activity status (MDA).

**Conclusion:**

In this large, real life, modern cohort of patients with PsA with frequent comorbidities who were treated mainly with biologics, almost half achieved minimal disease activity. These results show the value of existing therapeutic approaches while at the same time highlight the existing unmet needs.

## INTRODUCTION

Psoriatic arthritis (PsA) is a chronic inflammatory arthritis belonging to the group of spondyloarthritides (SpA) that is characterised by peripheral arthritis (with different patterns, oligoarthritis or polyarthritis -symmetrical or asymmetrical) and skin psoriasis (PSO). Other musculoskeletal manifestations such as enthesitis and dactylitis are quite common, while axial disease (AxPsA) is encountered in 20–40% of PsA patients.^1,2^ To add another level of complexity, extra-musculoskeletal manifestations, like inflammatory bowel disease (IBD) and uveitis are more common in PsA compared to the general population. Finally, an important aspect of PsA that often affects therapeutic decisions are the comorbidities associated with the disease, the most common belonging to the cardiometabolic (eg, diabetes mellitus, cardiovascular disease) and mental health (eg, depression, anxiety) spectrum.^3,4^ Considering the above-mentioned heterogeneity and also genetic and environmental factors, as well as the recent introduction of novel targeted treatments for PsA, disease and patient characteristics might vary between geographical areas^5^ and also over time. Herein, we aimed to describe the current demographic, clinical and therapeutic characteristics of PsA patients in Greece based on data prospectively collected from a nationwide, multi-centre, large patient cohort.

## METHODS

Under the auspices of the Greek Rheumatology Society (ERE-EPERE), a working group for PsA was formed, in which, rheumatologists from University Hospitals, from National Health System Hospitals, as well as from private practices participated. Subsequently, a protocol was agreed among the members of the group. According to that, data from all patients that attended the clinics/practices between 1/1/2022 and 31/12/2022 were recorded (baseline visit). Follow up data from the same patients will be recorded and at 1 year and at 3 years after the baseline visit.

In an electronic platform, the following characteristics were recorded. Domain 1 (demographics): gender, weight, body mass index, working status [employed, unemployed, pensioner], educational status [primary, secondary, university/college], time from the onset of symptoms to disease diagnosis, time of follow-up [time from disease diagnosis to baseline visit], Domain 2 (disease clinical characteristics): date of diagnosis, family history [of psoriasis, PsA, axial spondyloarthritis (AxSpA), and of IBD], as well as musculoskeletal manifestations [type of peripheral arthritis, axial disease-defined as symptoms of inflammatory back pain accompanied by radiologic findings in X-rays or MRI-, enthesitis, dactylitis] and occurrence of nail disease, IBD and uveitis at disease diagnosis, and/or throughout disease course. Clinical and laboratory characteristics [at the time of baseline visit as well as at the time of disease diagnosis], including number of tender and swollen joints, enthesitis assessed by Leeds enthesitis index (LEI), psoriasis assessed by body surface area (BSA), visual analogue scale (VAS) for patient’s global assessment for disease activity, VAS for patient’s global assessment for pain, erythrocyte sedimentation rate (ESR), C-reactive protein (CRP), health assessment questionnaire (HAQ), disease activity in PsA (DAPSA), as well as ankylosing spondylitis disease activity score (ASDAS) and Bath ankylosing spondylitis disease activity index (BASDAI), where applicable, were recorded. Domain 3 (Imaging): last available Magnetic resonance-MR for sacroiliac joints, cervical, thoracic, or lumbar spine. Assessment of Spondyloarthritis International Society (ASAS) definitions for positive MRI of the Sacroiliac joints or the spine were used.^6,7^ Last available X-rays of the spine (cervical, thoracic, lumbar) and of hands were recorded. Presence of syndesmophytes and of erosions/new bone formation, respectively was documented. Domain 4 (comorbidities): dyslipidaemia: cholesterol>200 mg/dl and/or low-density lipoprotein >130 mg/dl and/or triglycerides>150 mg/dl and/or receiving lipid-lowering therapy; coronary artery disease (CAD): myocardial infarction or angina or history of coronary revascularization; diabetes mellitus: treatment with anti-diabetic drugs; hypertension: blood pressure >140/90 mmHg in two measurements or treatment with antihypertensive medications; stroke (ischemic or haemorrhagic); chronic obstructive pulmonary disease; osteoporosis: bone density Tscore −2.5 or less in dual-energy X-ray absorptiometry or anti-osteoporotic treatment; depression: treatment with anti-depressants prescribed by a psychiatrist; hyperuricemia: serum urate>7mg/dl or treatment with uric acid lowering therapy; gout: history of gout episodes; neoplasias: current or previous. Domain 5 (Infections): Hepatitis B Virus status (HBsAg, Anti-HBs, Anti-HBc), Hepatitis C Virus status (anti-HCV, HCV-RNA), Tuberculosis (tuberculin skin test-TST and quantiferon test status, treatment for latent tuberculosis), Herpes zoster history, history of vaccination against flu and *str. Pneumoniae*, Infection that required hospitalisation during the last year, Domain 6 (Treatment): current treatment, previous treatments [and reason for discontinuation]; all approved non-biologic, targeted synthetic agents and biologics were considered. For glucocorticoids, mean dose of prednisolone or equivalent for the last month was recorded. Finally, current treatment with non-steroidal anti-inflammatory drugs (NSAIDs) was recorded.

Ethical approval was provided by the local institutional boards of participating centres and informed consent was provided by all patients before their inclusion in the study.

Statistical analysis was conducted using GraphPad Prism 5.00 (GraphPad Software, Inc., USA) and SPSS 24.0 (SPSS software, USA). Categorical features were compared with two-sided Fisher’s tests. Statistical significance is considered for *p-*values less than 0.05.

## RESULTS

### Demographics

In total 923 patients (55% females) with a median (IQR) age of 57 (48–65) years were enrolled in the study (**Table 1**). Their mean (SD) BMI was 29.1 (5.9) kg/m^2^ and 28.7% of them were smokers. The mean (SD) disease duration since diagnosis was 9.5 (8) years. Family history of psoriasis or PsA was noted in 28.3% and 6.3%, respectively (**Table 1**).

**Table 1.** Demographic, socioeconomic, and disease characteristics of patients with psoriatic arthritis.

**Characteristics**	**n=923**
**Demographic**	**n (%)**
Gender (female), n (%)	508 (55.0)
Age, median (IQR)	57 (48–65)
Age at Diagnosis, mean (SD)	47 (13)
Smoking current, n (%)	251/875 (28.7)
Weight, mean (SD)	84 (18.6)
Height, mean (SD)	169.8 (9.4)
BMI, mean (SD)	29.1 (5.9)
Delay in Diagnosis, years, median (IQR)	1 (0–3) ^*^
**Employment status**	**n (%)**
Employed, n (%)	553/886 (62.4)
Unemployed, n (%)	82/886 (9.3)
Retired, n (%)	251/886 (28.3)
**Education level**	**n (%)**
1^st^, n (%)	105/852 (12.3)
2^nd^, n (%)	484/852 (56.8)
3^rd^, n (%)	263/852 (30.9)
**Family history**	**n (%)**
Psoriasis	261 (28.3)
Psoriatic arthritis	58 (6.3)
Axial Spondyloarthritis	23 (2.5)

**Clinical Features**	**n (%)**	
	**At diagnosis, n (%)**	**Ever, n (%)**
Peripheral arthritis	710 (76.9)	868 (94.0)
Monoarthritis	39/640 (6.1)	
Oligoarthritis	248/640 (38.6)	
Polyarthritis	352/640 (55.2)	
Axial disease	143 (15.5)	239 (25.9)
Enthesitis	240/888 (27)	429/888 (48.3)
Dactylitis	176/888 (19.8)	295/888 (33.2)
Nail disease	281/877 (32.0)	377/877 (43.0)
Uveitis	12/864 (1.4)	28/864 (3.2)
Ulcerative colitis	3/871 (0.4)	7/871 (0.8)
Crohn’s Disease	4/871 (0.5)	14/871 (1.6)

IQR: Interquartile range; n: number; SD: standard deviation;

* n=780, from the times of symptoms onset to the time of disease diagnosis.

### Clinical characteristics

In our cohort, psoriasis preceded or was concurrent with the diagnosis of PsA in 66.7% and 15.9% of the patients respectively, while in 17.4% arthritis occurred before psoriasis. The median (IQR) time to PsA diagnosis since musculoskeletal symptom onset was 1 (0–3) years.

At diagnosis, peripheral arthritis was present in ∼70% of patients, with the most common pattern being poly-arthritis (55.2%) followed by oligoarthritis (38.6%) and monoarthritis (6.1%). AxPsA was present in 15.5% of patients, while enthesitis, dactylitis and nail involvement were reported in about one third of patients (**Table 1**). Extra-musculoskeletal manifestations such as uveitis and IBD, were uncommonly reported at diagnosis (<1%). During the disease course (from diagnosis to first evaluation) almost all (94%) patients had developed peripheral arthritis, while the rate of axial involvement increased to 25.9%. A similar increase in the rate of enthesitis, dactylitis and nail disease was also observed reaching 48.3%, 33.2% and 43.0%, respectively. Finally, the proportion of uveitis and IBD remained low throughout the disease course (<5%, **Table 1**).

### Comorbidities

**Figure 1.**
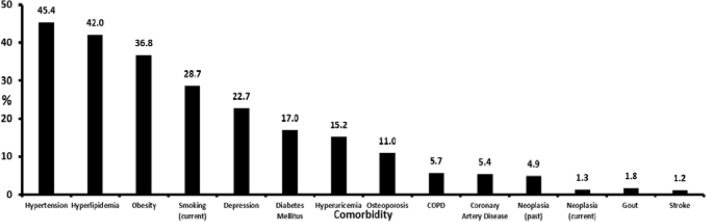
Graphical representation of patients’ comorbidities.

Comorbidities were present in most patients in our cohort with a median (IQR) number of 1^2^ comorbidity per patient. As expected, most of them belonged to the spectrum of cardiometabolic diseases, with 6.6% having CAD or stroke, 17% type 2 diabetes while about half of them had hypertension and/or dyslipidaemia (**Figure 1**).

In relation to that, hyperuricemia and obesity were also quite commonly observed. Of note, about one fourth of patients were treated for symptoms of depression. Finally, about 5% of the patients had a history of neo-plastic disease (**Figure 1**).

**Figure 2.**
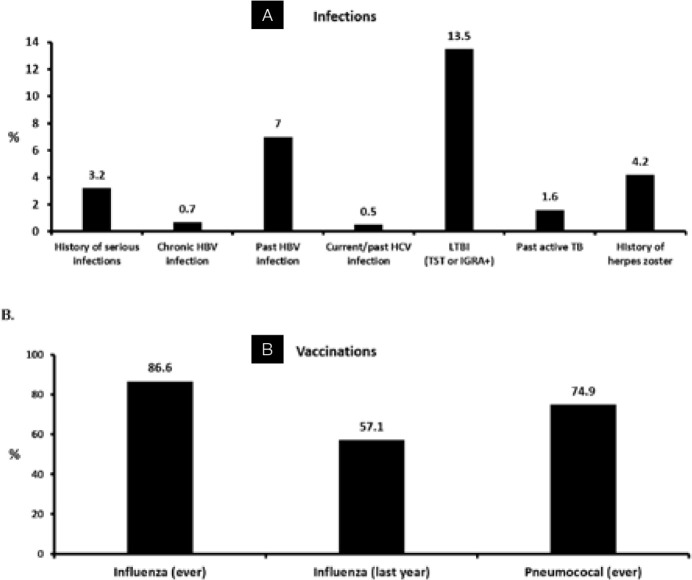
History of infections (A) and vaccinations (B) of the patient cohort. LTBI: latent tuberculosis; HBV: hepatitis B virus; HCV: hepatitis C virus; TST: tuberculin skin test; IGRA: interferon release gamma assay.

Regarding infections, chronic or past HBV infection was seen in 0.7% and 7% of the patients, respectively, while the frequency of current or past HCV infection was <0.5% (**Figure 2A**). Latent tuberculosis (LTBI) as defined by a positive TST or IGRA test was present in 13.5% of the patients while past active TB was reported by 1.6% of the patients. Finally, history of serious infections the previous year was recorded for 3.2% (29/911) of the patients. More than half of patients had been vaccinated against influenza the preceding year (57.1%) while the majority had been vaccinated against flu or *Streptococcus pneumoniae* in the past (86.6% and 74.9%, respectively, **Figure 2B**).

### Treatment characteristics

**Figure 3.**
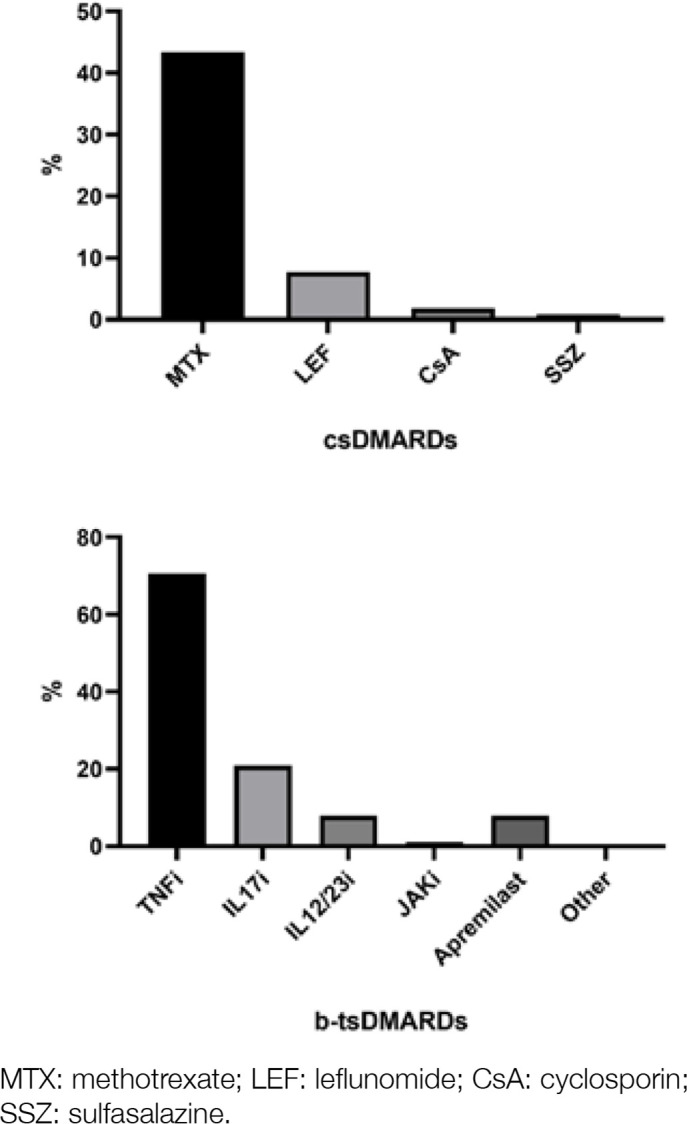
Treatment characteristics of included patients. (a) Non-biologics, (b) biologics/targeted synthetic agents.

At the time of assessment (baseline visit), treatment with conventional non-biologic agents was reported for 53.6% of patients (**Figure 3A**), the most common being methotrexate (43.4%) at a mean (SD) dose of 14.1 (3.9) mg/week, followed by leflunomide (7.7%), cyclosporine (1.8%), and sulfasalazine (0.8%). 25.4% were on non-biologics alone. NSAIDs and glucocorticoids [mean (SD) dose: 5.6 (3.2) mg/day of prednisolone or equivalent) were received on a regular basis by 2.9% and 10.6% of the patients, respectively. Apremilast was used by 7.9% of the patients and JAK inhibitors (tofacitinib or upadacitinib) by 1.1% (**Figure 3B**).

More than half (60.1%) of patients were on biologics at the time of initial evaluation (**Figure 3B**); 70.6% were on tumour necrosis factor inhibitors (TNFi) while 28.8% were treated with anti-interleukin (IL)-12/23 or anti-IL-17 drugs (20.9% on anti-IL-17 and 7.9% on anti-IL-12/23). Among patients treated with biologics, 52.1% were receiving them as monotherapy. More patients treated with anti-IL-17 or-12/23 agents received them as monotherapy (64.2%) compared to those on TNFi monotherapy (49.4%, p=0.0001).

**Figure 4.**
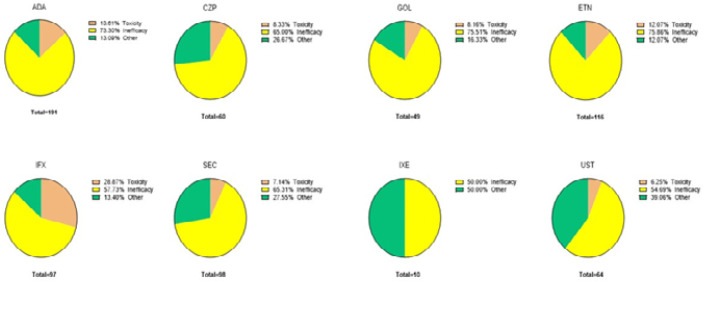
Biologic treatment utilisation and reason for discontinuation. bDMARDs received from patients at the time of baseline visit and reasons for discontinuation. ADA: adalimumab; CZP: certolizumab; GOL: golimumab; ETN: etanercept; IFX: infliximab; SEC: secukinumab; IXE: Ixekizumab; UST: ustekinumab.

Regarding previous biologic use, most of them were discontinued due to inefficacy, while drug-related adverse events leading to drug discontinuation ranged from 6.25% for ustekinumab to 28.87% for Infliximab (**Figure 4**).

### Disease activity

At the initial cross-sectional evaluation (baseline visit), the median PsA disease activity assessed by the DAPSA score was 6 (IQR: 2.3 – 13.1). The mean ESR and CRP values were 15.5 (14.1) mm/h and 0.98 (1.72) mg/l, respectively. Most patients had minimal psoriatic skin involvement (BSA<3: 82.6%), while 15.1% and 2.4% displayed BSA involvement of 3-10% and >10%, respectively. Moreover, minimal disease activity (MDA) (8) was recorded in 46% of the patients.

## DISCUSSION

In this large, nationwide, contemporary, cohort of patients with PsA we present a complete profile of their demographic and clinical features (including their extra-musculoskeletal manifestations and co-morbidities) as well as their current treatment patterns. Our study provides an accurate picture of the disease activity and rate of achievement of the treatment goals in the modern therapeutic era with non-biologic, biologic and targeted synthetic agents available.

The demographics of this patient cohort were similar to those reported in other real-world studies, ie, mostly middle-aged patients with no strong sex predilection.^9,10^ Interestingly, in about 40% of patients, there was a family history of an immune-mediated disease in the spectrum of PsA/SpA. Family history of axSpA or IBD was relative rare, while family history of psoriasis was the most common (28.3%), which is in agreement with findings from a similar cohort from Turkey,^11^ and highlights the common genetic and pathogenetic pathways operating at both PsA and psoriasis.^12^

Regarding their clinical characteristics, enthesitis and dactylitis were present in 20-30%^9,10,13–14^ and axial disease in ∼15% (15) of patients at diagnosis. It is worth noting that the proportion of patients affected by enthesitis or axial disease, doubled in the meantime (mean interval: 9.5 years) between PsA diagnosis and initial study (baseline) visit. Psoriasis did not seem to be a major problem in our cohort, with the majority of patients having a BSA <3%, although data about psoriasis severity at diagnosis were not available. In a study of patients with early PsA, Kasiem et al recently reported that psoriasis was severe in <5% of patients (16) while other studies had also shown that psoriasis appears to be mild in most PsA patients (9). Uveitis and IBD were also uncommon in our cohort (<5%), in accordance with recent studies.^17,18^

As discussed extensively in the literature and has been shown in smaller-scale studies,^3,19-21^ comorbidities are an important aspect of the disease with a considerable number of patients having concurrent cardiometabolic conditions. This is partly attributed to the disease inflammatory burden,^22^ as well as to relevant risk factors, like obesity, hypertension, dyslipidaemia, and diabetes.^19^

In our study, almost half of our patients had hypertension (45%) or hyperlipidaemia (42%), 37% were obese and 17% had type 2 diabetes. Compared to a recent cohort of 2491 patients with RA from the same country, patients with PsA, despite being younger (mean age: 57 *vs.* 63 years), had similar rates of hypertension (45% *vs.* 42%) and type 2 diabetes (17% *vs.* 15%) but more often hyperlipidaemia (42% *vs.* 33%) and obesity (37% *vs.* 26%) (23). Similarly, in a recent systemic literature review a high rate of metabolic syndrome in PsA was reported (23.5-62.9%).^24^

Another interesting finding of our study was that 15% of our patients had hyperuricemia and 1.8% gout, which is an underrecognised comorbidity in the setting of PsA. Although it appears that there is a close association between PsA and uric acid levels,^25^ less than a handful, relatively small studies have investigated this matter thus far,^26,27^ and found an association of hyperuricemia with body mass index (BMI) and with surrogate markers of cardiovascular disease. Besides, it has been shown that controlling for traditional cardiovascular risk factors might be the best strategy for reducing cardiovascular risk in PsA.^28^

Mental health disorders are also well-recognised comorbidities in the setting of PsA^20^ with the frequency of depression in a recent meta-analysis reaching 20%^29^ which is quite similar to our findings (22.7%). Studies from our and other countries have shown that these characteristics can negatively affect therapeutic effects^30–32^ and therefore should be actively sought and treated.

Screening and appropriate monitoring for serious and opportunistic infections in patients with inflammatory arthritides, especially in those receiving biologic or targeted synthetic therapies, has become the standard of care in daily practice.^23^ Data regarding their frequency in newer patient cohorts are limited. In our PsA cohort, the frequency of chronic and past HBV infection was slightly lower to that reported in a similar RA cohort from the same population (0.7% and 7% compared to 2.1% and 10%, respectively) while the prevalence of HCV infection (0.5% *vs.* 0.7%), LTBI (13.5% *vs.* 13–15.3%) and herpes zoster (4.2% *vs.* 6.2%) was similar between both cohorts.^23^ An interesting finding, that could be explained in part by the younger age of PsA patients and the less frequent use of glucocorticoids, was the lower history of serious infections in the PsA compared to the RA cohort (3.2% *vs.* 9.6%). This is an important finding that should be considered when making therapeutic decisions in this patient population.

An encouraging finding was the high rates of vaccination against influenza and pneumococcus in this patient population. The rates of influenza (86.6%) and pneumococcal (74.9%) vaccination in 2022 in PsA patients was much higher than the respective rates in an RA population from the same country in the years 2015–16 (52% and 36% respectively). The increased education and awareness of the rheumatology community for the benefits of vacci-nation, as well as the Covid-19 pandemic,^33^ most likely contributed to these increasing rates.

The proportion of patients who were receiving biologic agents in our cohort was 60% which is comparable to similar real-life cohorts.^9,13^ Most of them were in TNF inhibitors followed by anti-IL-17 and anti-IL-12/23 agents. In our cohort most of the patients receiving biologics were on monotherapy in agreement with data published in one of the largest studies on the field.^14^ In addition, we show for the first time in a real-world study, that biologic monotherapy was more commonly utilized by patients on anti-IL-17 or 12/23 agents than those receiving TNFi, the latter being about 50%, close to the proportion published by the EuroSpA collaboration few years ago.^34^ As regards safety, in our study ustekinumab and infliximab displayed the lowest and highest frequency, respectively, for discontinuation due to adverse events. This is in line with recent metanalyses in which IL-23 inhibitors demonstrated the lowest rates of infections^35^ and infliximab the highest rates of discontinuation due to adverse events in patients with PsA.^36^

Finally, regarding the achievement of the treatment goals in this real-life cohort, as these are set by the DAPSA score,^37^ the PsA disease activity was in the low/medium disease activity range for most of our patients. Furthermore, regarding the overall control of the psoriatic disease, approximately half of the patients reached MDA. These findings, which are close to those reported in other real-world studies^9^ and a recent meta-analysis,^38^ highlight the success of the newly introduced therapeutic agents and current management approach while at the same time emphasise the need for more efficacious treatment strategies in PsA.^9,39^

We acknowledge that our study has certain limitations. First, most of the patients were followed up in hospitals rather than in private practices. Thus, more severe cases are possibly over-represented. On the other hand, these hospitals were not necessarily tertiary referral centres for PsA and our patients were consecutively enrolled. Second, comorbidities were reported based on history and treatment received and not on specific questionnaires. However, percentages of comorbidities herein, are similar to those described in other studies, specifically designed for this purpose.^21,30^ Also, newer drugs (eg, IL-23p19 inhibitors) were not available for the treatment of PsA, at the time this study was conducted. These are expected to be captured at the subsequent time-points of this study (υear 1 and year 3). Finally, there are some missing data in some of the parameters recorded. These are within the acceptable 10% range for most of the cases.^40^

On the other hand, the strengths of our study include that it is a real-world nationwide study with data that are prospectively collected in a prespecified electronic form. Additionally, it is one of the largest studies in the field, designed to capture the heterogeneity of PsA in terms of demographic, clinical and therapeutic characteristics of patients.

In conclusion, our large cross-sectional study with over 900 patients with PsA, provides a modern capture of the overall disease profile and achievement of treatment goals. Regarding disease characteristics, the study confirmed that the extent of psoriasis is relatively limited in PsA patients. The most common pattern of joint involvement since diagnosis is polyarthritis while axial involvement affects gradually a significant proportion of patients. Co-morbidities were common, with hyperlipidaemia and obesity remaining a significant issue in this patient population. Serious infections appeared to be less common compared to synchronous RA cohorts while we observed an increasing vaccination rate in these patients. With the current management approach, which included the frequent use of biologics, most patients achieved low joint activity with almost half of them reaching the hard end point of minimal overall disease activity. These findings, by providing an accurate picture of psoriatic disease today, set the stage for designing and implementing the appropriate diagnostic, monitoring, and management strategies for the future.
